# Type 2 Myocardial Infarction Resulted from the Left Thoracic Stomach

**DOI:** 10.21470/1678-9741-2019-0208

**Published:** 2020

**Authors:** Yaming Shi, Yongzhong Zong

**Affiliations:** 1 Department of Cardiology - The Third People’s Hospital of Yancheng, Jiangsu Province, Yancheng, China.

**Keywords:** Myocardial Infarction, Type 2, Myocardial Infarction, Inferior Wall Myocardial Infarction, Stomach, Dilatation

## Abstract

The universal definition of myocardial infarction (MI) provides five subtypes of acute myocardial infarction (AMI). We present an interesting case of a type 2 myocardial infarction caused by the dilation of the left thoracic stomach.

**Table:** 

Abbreviations, acronyms & symbols	
AMI	= Acute myocardial infarction
aVF	= augmented voltage foot
aVL	= augmented voltage left arm
CAD	= coronary artery disease
ECG	= Electrocardiogram
LTS	= left thoracic stomach
LVH	= left ventricular hypertrophy
MI	= Myocardial infarction
ST	=ST segment

## INTRODUCTION

The universal definition of myocardial infarction (MI) provides five subtypes of acute myocardial infarction (AMI). We present an interesting case of a type 2 myocardial infarction caused by the dilation of the left thoracic stomach.

## CASE REPORT

**Fig. 1:**
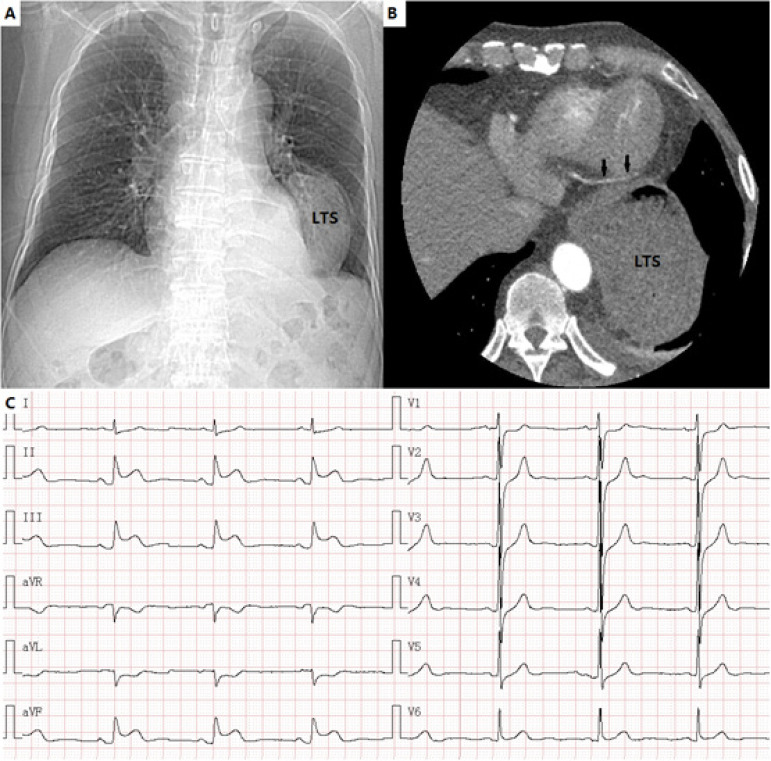
Images of chest radiography, computerized tomography angiography and ECG. A) Chest radiography revealed left thoracic stomach; LTS=left thoracic stomach; B) The distal right coronary artery coursed between the left ventricle and the thoracic stomach (arrows). C) Standard 12-lead ECG revealed sinus bradycardia with ST segment elevation in leads II, III and aVF and ST depression in leads I, aVL.

A 75-year-old man was presented with a six-month history of chest pain radiating to the arms, which worsened after meals. He had cancer of the gastric cardia and underwent an esophagectomy with gastric pull-up in 2010. Physical examination was unremarkable, except for a scar from his previous operation. Initial standard 12-lead electrocardiogram (ECG) showed sinus bradycardia with no evidence of ST segment abnormalities. Chest radiography revealed left thoracic stomach (Figure 1A). Transthoracic echocardiography showed normal left ventricular dimensions and 60% ejection fraction. Computerized tomography angiography demonstrated that there was no evidence of right or left coronary artery stenosis, with the distal right coronary artery coursed between the left ventricle and the thoracic stomach (Figure 1B, arrows). On the second day, an endoscopic examination showed gastric retention without gastric outlet obstruction. On the same day, after eating more than usual, the patient complained of chest pain during 30 minutes, associated with diaphoresis. Standard 12-lead ECG was performed again, which revealed sinus bradycardia with ST segment elevation in leads II, III and aVF and ST depression in leads I, aVL (Figure 1C). Laboratory tests showed an increase of cardiac troponin I (6.26ng/ml; RV < 0.3ng/ml). At this point, it was recognized that the distal right coronary artery might be the culprit infarct-related artery. However, urgent coronary angiography did not show angiographic evidence of a thrombus. The case was discussed in an emergency consultation between the cardiologists and the gastroenterologists. The patient was diagnosed with a type 2 myocardial infarction with gastroparesis. Therefore, a decision was made to relieve his symptoms with a motilin agonist, and the patient was also advised to cut down on the amount of his meal. The patient was discharged home with mosapride and remained asymptomatic at the follow-up.

## DISCUSSION

The diagnosis of type 2 MI has been examined in a large Swedish registry study^[1]^. In all, 20138 hospital patients with a diagnosis of AMI were examined. The majority of cases (88.5%) were classified as type 1 MI, while 7.1% classified as type 2 MI. Type 2 MI is considered to have taken place: in instances of myocardial injury with necrosis were a condition other than CAD contributes to an imbalance between myocardial oxygen supply and/or demand *e.g*. coronary endothelial dysfunction, coronary artery spasm, coronary embolism, tachy-/brady-arrhythmias, anaemia, respiratory failure, hypotension, and hypertension with or without LVH^[2]^. We reported a case of type 2 MI caused by the dilation of the thoracic stomach. The distal right coronary artery was compressed by the dilation of the thoracic stomach, and that may result in inferior wall myocardial infarction. We recommend that the cardiologists and the gastroenterologists be aware that inferior wall myocardial infarction can be caused by the dilation of the left thoracic stomach.

**Table:** 

Author's roles & responsibilities	
YS	Substantial contributions to the conception or design of the work; or the acquisition, analysis, or interpretation of data for the work; drafting the work or revising it critically for important intellectual content; final approval of the version to be published
YZ	Substantial contributions to the conception or design of the work; or the acquisition, analysis, or interpretation of data for the work; drafting the work or revising it critically for important intellectual content; final approval of the version to be published
